# Comparative genomics of *Helicobacter pylori *isolates recovered from ulcer disease patients in England

**DOI:** 10.1186/1471-2180-5-32

**Published:** 2005-05-25

**Authors:** Farhana Kauser, M Abid Hussain, Irshad Ahmed, Sriramula Srinivas, S Manjulata Devi, Ahmed A Majeed, K Rajender Rao, Aleem A Khan, Leonardo A Sechi, Niyaz Ahmed

**Affiliations:** 1 Pathogen Evolution Group, Laboratory of Molecular and Cell Biology, Centre for DNA Fingerprinting and Diagnostics (CDFD), Hyderabad, India; 2 ISOGEM Working Group on Genetics of Helicobacters [International Society for Genomic and Evolutionary Microbiology (ISOGEM)], Hyderabad, India; 3 Deccan Medical College and Allied Hospitals, Hyderabad, India; 4 Department of Biomedical Sciences, University of Sassari, Sassari, Italy

## Abstract

**Background:**

Genomic diversity of *H. pylori *from many different human populations is largely unknown. We compared genomes of 65 *H. pylori *strains from Nottingham, England. Molecular analysis was carried out to identify rearrangements within and outside the *cag*-pathogenicity-island (*cag *PAI) and DNA sequence divergence in candidate genes. Phylogenetic analysis was carried out based on various high-resolution genotyping techniques.

**Results:**

Analyses of virulence genes (*cag*T, *cag*E, *cag*A, *vac*A, *ice*A, *oip*A and *bab*B) revealed that *H. pylori *strains from England are genetically distinct from strains obtained from other countries. The toxigenic *vac*A s1m1 genotype was found to be less common and the plasticity region cluster was found to be disrupted in all the isolates. English isolates showed a predominance of *ice*A1 alleles and a functional proinflammatory *oip*A gene. The English *H. pylori *gene pool revealed several Asian/oriental features. This included the predominance of *cag*A – *glr *(*cag*A right junction) motif types III and II (up to 42%), presence of *vac*A m1c alleles and phylogenetic affinity towards East Asian / Amerindian gene pools based on fluorescent amplified fragment length polymorphism (FAFLP) analysis and *glm*M sequence analysis.

**Conclusion:**

Overall, our results demonstrated genetic affinities of *H. pylori *in England with both European and the Asian gene pools and some distinctive genetic features of virulence genes that may have evolved in this important European population.

## Background

Infection of the gastric mucosa with *H. pylori *results in a number of disease outcomes including gastritis, which precedes the development of peptic ulcer disease, gastric cancer and lymphomas of the MALT [1-3]. These diseases caused by *H. pylori *and their prevalence rates differ in different geographic countries and only a subset (10%) [4] of infected patients develop one of them. This raises the question as to why *H. pylori *causes disease in a few individuals, but not in the great majority [5].

Many studies have demonstrated the involvement of bacterial virulence factors, host genetics and environmental factors in contributing to the development of disease. Bacterial virulence factors include proteins mediating establishment/colonization, persistence of infection and finally long-term damage to the host [6]. The *cag *pathogenicity-island (*cag *PAI) is the most noteworthy among these factors. PCR analyses have suggested that this island is not intact in many strains across the world [7] and the presence of an intact PAI although not always [8,9] is indicative of a more severe outcome [10]. The expression of various products encoded in the *cag *PAI is known to be involved in inducing inflammation, ulceration and carcinogenesis [11]. However, the *cag*A is expressed by the majority of *H. pylori *strains, irrespective of the geographic origin and clinical diagnosis [12].

The vacuolating cytotoxin antigen (VacA) is another virulence factor that is considered to constitute an increased risk for development of peptic ulcers and gastric cancer [13,14]. Allelic variations in the *vac*A gene are found in the signal (s1, s2) and the middle region (m1a, m1b, m2) and the s1 type is associated with ulcer disease [14,15].

More pronounced inflammation is associated with strains, which express the outer membrane protein OipA. OipA induces IL-8 secretion by epithelial cells. Active OipA protein production may be 'on' or 'off' depending on the number of CT repeats in the signal sequence of the *oip*A gene (HP0638). *H. pylori *strains may also be grouped geographically based on *oip*A sequence pattern [16]. Specific adhesins viz., *bab*A and *bab*B mediate the adherence of the bacterium to specific human blood group Lewis antigens and are associated with various disease outcomes [17]. Similarly, a putative *E. coli *restriction enzyme *Nla*III homologue, the *ice*A gene in *H. pylori*, which is activated on contact with the epithelium, is also shown to induce high levels of IL-8 [18].

Accordingly, strains with *Oip*A 'on' status, active forms of *ice*A and *bab*A [18] and particularly strains which are *cag*A+ and *vac*A s1 have been shown to cause a more severe outcome [14,15,19], though not in all cases [20].

Many studies have pointed out a bio-geographical variation in virulence factors; for example, the sequences of *vac*A and *cag*A differ in strains from the United States and Europe from those in China and Japan [21]. Also, the prevalence and type of *H. pylori *infection varies with a very high rate of occurrence (up to 70%) in Asia and the Middle East [22], in contrast to only 30–50% in Europe and the United States [23]. Further, the infection is minimal in children in the west while in the rest of the world it affects both young and the old. Active infection with *H. pylori *was seen in about 7.5 million people in the general population of England and Wales. This although varied from one region to the other with the highest rates recorded in London [24]. Thus *H. pylori *remains an important infection in the UK.

*H. pylori *population has been described as highly recombining, and therefore exhibits enormous strain diversity, part of it may be due to the presence of the plasticity zones [25]. Since this organism has also been shown to be transmitted within families, a greater number of epidemiological studies reveal that these strains not only show similar genotypic profiles when obtained from related patients but also show common profiles within isolates from specific countries [26]. Phylogeographic affinities were pronounced in case of European strains based on the multi locus sequence typing of seven housekeeping genes where the European strains and the Asian strains shared an ancestral relationship [27]. This observation was also recorded in other studies based on the evolutionarily conserved ERIC sequences that indicated close associations between the Irish, Spanish and the European strains and also clustering of the English strains with a few Asian strains [26]. However, the number of English strains used in that study was very small. It is noteworthy in this respect that comprehensive phylogenetic analyses in case of English strains have been rarely performed.

In this study, we aimed at a comprehensive assessment of the prevalent genetic structure of *H. pylori *strains infecting the English population in Nottingham, which is centrally located in the United Kingdom. The strains were analyzed to study a total of 45 different parameters pertaining to 28 informative loci including the virulence factors *cag*A, *oip*A, *ice*A and *vac*A in addition to other genes of the *cag *PAI and the motifs downstream to it. Composition of the plasticity region cluster including the putative gastritis (JHP0986) and gastric cancer (JHP0947) associated markers were also studied [25]. Phylogenetic analyses were performed using FAFLP markers, nucleotide sequences of the *cag*A, *bab*B and the *glm*M genes and the repetitive sequences interspersed in the *H. pylori *genome (ERIC and REP). According to our observations, phylogenetic placement of English strains shows affinities with East Asian and Amerindian strains.

## Results

**Figure 1:**
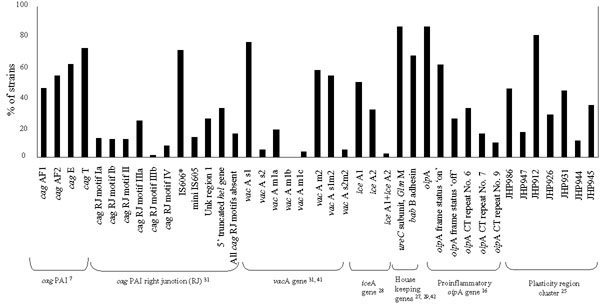
Diagram showing the percent distribution of different genetic loci in the English isolates. The presence of the *cag *PAI with the motifs on its right end, the *vac*A and *ice*A genotypes and the presence of the *glm*M and *bab*B house keeping genes and characteristics of the virulence gene *oip*A and the genes included in the plasticity region cluster are shown. The distribution was determined by PCR using primer sequences from the reference articles denoted by the number in superscript.

**Figure 2:**
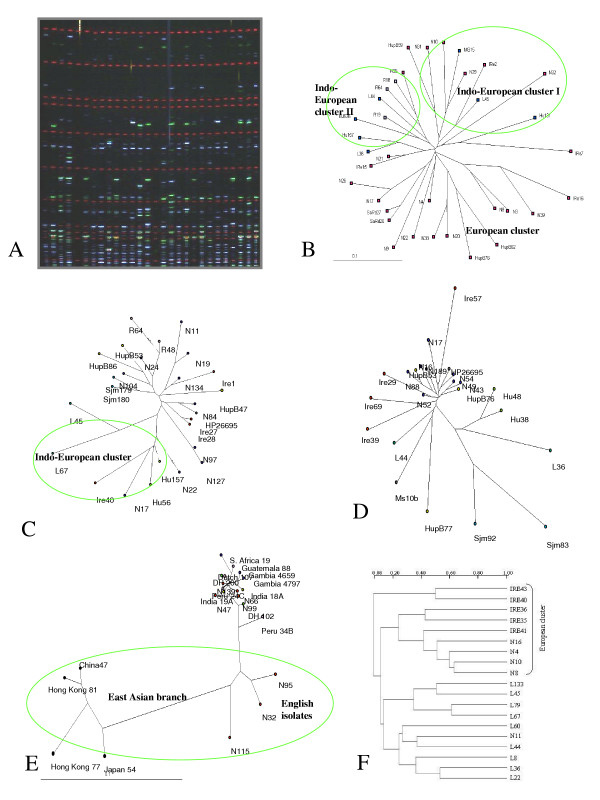
(A) shows a gel image of representative FAFLP patterns of English *H. pylori *strains with the bands in red indicating the internal lane standards (Genescan Rox 500). (B) Representative phylogenetic tree indicating genetic relationships between English *H. pylori *strains (n = 16) and those from other world populations (HupB, n = 3; Ire, n = 3; Italy (Sard), n = 2; R = 2; E. Asia (Hu), n = 2; India (L, MS, BJ), n = 5) based on the FAFLP profiles generated for the strains from different countries (described in the materials and methods section). The neighbour joining network tree created based on the level of similarity between the amplitypes shows that European strains form a distinct cluster although two other clusters showing associations with the Indian strains are also seen. (C) Cluster analysis of the 296 bp *glm*M was generated with 10 English strains, 2 strains each from India (L), E. Asia (Hu), Peru (Sjm) and S. Africa (R). 4 each from Ire and HupB and the sequenced HP26695. The rounded circle here shows the grouping of English islolate (N 17) with the Indian (L) and E. Asian (Hu) strains. (D) Tree constructed from the *bab*B gene products from different strains (N, n = 8; Ire, n = 4; HupB and MS, L, n = 3 respectively, 2 each of Hu and Sjm with HP26695). (E) Genetic relationships of the English strains based on the 250 bp sequence of the 5'end of the *cag*A gene (England, n = 7; East Asia, n = 4; S. Africa, n = 1; W. Africa, n = 2; S. America, n = 2; central America, n = 1; India, n = 2; Bangladesh, n = 2 and Holland, n = 1). A few strains from Nottingham showed sequence similarity with the Asian strains as indicated by the circles in green. (F) Phylogenetic tree based on the REP fingerprinting patterns generated by the English strains (n= 5) in comparison with 5 strains from Ire and 6 from India (L).

Details of all the genotyping and phylogenetic analyses have been depicted in Figures 1 and 2 and are summarized in Additional file 1.

### Macroscale analysis of the *cag*-PAI and the downstream motifs

The status of *cag *A gene was assessed by using primers specific to sequences at both the 5' end and the 3' ends. PCRs for the 5' end were positive in 41 strains (62.1 %), whereas only 35 strains (53%) were PCR positive for the *cag *3' end. Twenty-one strains (31.8%) had both the ends detected by PCR indicating therefore the possible presence of a complete gene.

Of the ten strains PCR negative for both *cag*A ends, the *oip*A gene frame status was "on" for 8 of them. Hence we showed no association between the presence of the *cag*A gene and the frame status of *oip*A. Only two strains out of the 10 completely PCR negative for *cag*A did not have any motif type on the right end of the gene PCR amplified.

The most frequently detected gene by PCR in UK strains was *cag*T (83.3%) followed by *cag*E (71.2%).

Upon analysis of the extreme right junction of the *cag *PAI (region extending from the *cag *A 3' region to the *glr *gene), 54 strains out of 66 had either the type IIIa motif (28.8%) or the type I (Ia/Ib) motif (28.8%). The type IIIb motif was observed in a single isolate (N3), while 13.6% strains displayed the type II motif. The type IV motif was amplified in only 9% of the strains and among the three strains recovered from patients of Indo – Pakistani origin, N105 showed a type I a signature, while N115 showed type II motif. The type III motif was also observed for strains from patients of Chinese (N 99) and Russian (N90) ethnicities settled in UK.

The frame status of the *oip*A gene was 'off' in 8 of the 12 strains that did not have successful amplification of any motif types on the right end of the *cag *PAI and between the 3' end of the *glr *gene.

The sequence of the 250 bp product amplified from the 3' end of the *cag*A gene was determined for 16 English strains. Phylogenetic analyses of these sequences in comparison with others from Holland (n = 1), East Asia (n = 4), India (n = 2), Bangladesh (n = 2), South America (Peru, n = 2 and Guatemala, n = 2), South Africa (n = 1) and Gambia (n = 2)) (Figure 2E) revealed that the Asian strains carried a unique *cag*A gene sequence and formed a segregated cluster. Strains from Africa clustered close to the Indian ones whereas English strains showed no specific clustering.

### *vac*A and *ice*A statuses of the isolates

87.8 % of the strains possessed the toxigenic type s1 *vac*A allele while the less toxigenic s2 allele was detected in ~ 6% strains. The *vac*A m2 genotype was present in 66.6% strains and only 21.2 % strains had the m1a genotype. The m1c subtype found in strains from India [28] was observed in 3 strains and none of the strains had a type m1b *vac*A allele. Therefore, the s1m2 type of *vac*A was most commonly (66.6%) found in these English strains. Only 38 of the 58 strains (65.5%) with *vac*A s1 allelic subtype had the *oip*A gene in frame.

The *ice*A1 allele was present in 38 strains (57%) whereas the *ice*A2 allele was found in 24 strains (36.3%). Only two strains were positive for both the alleles (N22, N105). The likely explanation is that these "strains" were in fact mixture of two strains.

### Status of the Proinflammatory protein *oip*A gene (HP0638)

Strains from UK mostly had the *oip*A frame status 'on' (70.5%) with the CT dinucleotides repeated 6 times in 37.7% strains. This was followed by the repeat number of 7 observed in 18 % strains and 9 in 11.5% strains. 10 CT repeats were found in a single isolate (N52) and a single repeat was shown in three strains. These results and those for other loci studied are shown in the bar diagram [Figure 1].

### Inventory of the plasticity region ORFs in English strains

The ORF HP986 (referred to as the gastritis associated marker) [25] was PCR amplified in 31 strains (52.5%), while the gastric cancer associated ORF JHP947 [25], was amplified in only 10 strains (19.2%). Other ORFs from this region that were amplified included JHP912, which was seen in 93.5% strains. The ORF JHP926 was amplified in 32.6 % of the strains while a J99 specific ORF JHP931 thought to be involved in DNA replication [25] was found in 51.1% strains. ORFs JHP944 and JHP945 were amplified in 13% and 40% strains respectively. None of the strains showed any specific pattern of ORFs within the plasticity region.

### Phylogenetic placement and affinities with other genogroups

The housekeeping gene, *glm*M, was present in all the strains (100%) and the adhesin *bab*B was amplified in 51 strains (77.27%). The *bab*B gene has been a marker of choice for tracing lineage in *H. pylori *and recent studies employing this gene have postulated *H. pylori *'s association with its human host to be approximately 11,000 years old [27,29]. Hence phylogenies [Figure 2C – *glm*M tree and 2D – *bab*B tree] were generated based on the sequences of these genes in representative strains. These phylogenies revealed that strains from England clustered with other European strains (Ireland-Ire and Spain-HupB), while some affinities between them and Peruvian strains could also be noticed. Individual branches representing geographically specific *glm*M sequences were observed for India (MS, L), Japan (Hu), and Africa (R).

FAFLP patterns of English strains revealed about 130 fragments in the size range of 50–500 bp when the genomic DNA was digested with enzymes MseI+0/EcoRI+A. A binary table indicating the presence or absence of a particular amplicon in each strain was scored and these values were used to assess the genetic relatedness within English (abbreviated N) and with other European strains including those from Sardinia (SarD), Spain (HupB) and Ireland (Ire). These strains clustered in one group labeled in the figure as the European cluster. Another cluster obtained was the one which represented contribution from Asian- European gene pool and included strains from India (L, BJ & MS), Japan (Hu), Africa (R) and others from Europe (HupB, Ire, N) [Figure 2B].

A similar trend was observed with other fingerprinting techniques employing the Enterobacterial Repetitive Intergenic Consensus sequences (ERIC) and the Repetitive Extragenic Palindrome (REP) sequences. Based on the amplification patterns of genomic DNA between the REP sequences, the English strains grouped with the Irish ones in a European cluster and a segregated cluster constituting all Indian strains from Ladakh and a single strain from England (N114) was obtained [Figure 2F]. ERIC phylogeny for this set of strains in comparison with other strains from different world populations as reported earlier [26] indicated that these strains clustered closely with either the Spanish and Irish strains and more distantly with the Indian and African strains.

## Discussion

Evolution of infectious microorganisms is a consequence of the genetic polymorphisms they accumulate, which in turn is the result of the long term selection pressure exerted by the host immune system in case of chronic infection as well as the environment [9,30]. This is more pronounced in case of bacteria like *H. pylori *that cause multi decade long infections, wherein an acquiescent requirement to constantly keep their genomic content recasting is crucial. In *H. pylori *this concern is resolved by the use of restriction – modification systems and regulation at gene level by nucleotide substitution, insertion and deletion events [31]. Further, the presence of insertion elements, plasticity regions and the pathogenicity islands contribute considerably to its genetic diversity.

We attempted to analyze genetic variation and structure of *H. pylori *populations infecting the native people of England. The UK today is more culturally diverse than ever before with the majority of the UK population being ethnic Europeans (92 %). The remaining 4.6 million (or 7.9 %) people represent other ethnic groups. South Asians are the largest of these groups, followed by Caribbean and Black Africans [32]. Such a multiethnic presence creates an interesting genetic conundrum when we attempt to analyze incidence and healthcare-impact of any pathogen that biases itself with respect to the host genetic makeup. Also, associations of the disease outcome with the virulence factors has thus far been enigmatic and since there were no comprehensive studies involving multiple loci for genotype-phenotype assessments, the current study was envisaged in combination to generate base line data relevant in molecular epidemiology of virulent strains in England.

The *cag *PAI is a major virulence determinant in *H. pylori *and strains lacking this island are akin to commensals rather than pathogens [1]. Reports suggest that the presence of a complete set of genes within the *cag *PAI ensures a 5-fold increased severity of disease outcome than the intermediate PAI [10]. We have earlier showed that a higher number of strains from Japan had an intact *cag *PAI [7], hence it may be thought as an important factor influencing the outcome of the infection as a higher rate of gastric cancers was observed in the Japanese patients. The possible role of *cag*A in oncogenic mechanisms is being worked upon [33]. Most English strains in our case retained the *cag*T and the *cag*E genes. Studies showed that strains lacking the *cag*T gene had a defective 'molecular syringe' that is encoded by the PAI [34] reflecting thus on inability of the Type IV system to eject out the *cag*A protein. The *cag*E gene on the other hand is known to induce NFkB activation and IL8 secretion [8] in addition to mediating host-cell cytokine rearrangements in infected epithelial cells. About 25 % of the strains carried the type IIIa motif on the right end of the *cag*A gene. This observation also supports the hypothesis that some of the European strains share some features typical of the Asian genogroups. This is also supportive of an earlier observation [31] that a very small number of strains from the European countries also show the type III motif.

English strains mainly showed a higher number of the toxigenic s1 type of *vac*A allele. Interestingly, the s1m1 combination was observed in less percentage in contrast to the s1m2 genotype that was seen in 60 % of the cases. Earlier studies in a subset of European population (Mid-Essex) by RFLP of the mid-region of the *vac*A gene in strains originating from dyspeptic patients demonstrated that 46% of these strains had the s1m2 genotype while 40% strains had the more toxigenic s1m1 combination [35]. The s1m2 genotype was also common in strains from North Wales among clarithromycin sensitive and resistant *H. pylori *[36]. It was interesting to find the m1c allele [28], which is known to be prominent in the East Asians, in UK strains. None of the strains had multiple *vac*A genotypes, which are common in China and other East Asian countries [37]. It has been reported earlier that the s1 allele was most frequently observed in the European population with the s1a allele predominant in Northern and Eastern Europe. Also, s1a and s1b alleles were observed in France and Italy while the Spanish and Portuguese strains had the s1b subtype [13].

The virulence spectrum of the English strains was also exemplified by the observation that 70% of these strains had the *oip*A gene in frame. Greater than 6 CT repeats in the upstream homopolymeric tail of the *oip*A gene is characteristic of European strains [16]. Our results indicate that English strains most commonly displayed 6 CT repeats. Strains with 9 CT repeats were reported to have the gene out of frame owing to the deletion of the CTAA sequence present immediately upstream of the CT repeats [16]. Similar results were found with English strains with 9CT repeats. From our analyses, the *ice*A1 allele was more common than the *ice*A2 allele. Although no association between *ice*A subtypes and clinical outcome has been reported, strains carrying *iceA1 *produce higher levels of IL-8 in the gastric mucosa and are more often associated with DU in the North America and Dutch people than strains carrying *iceA2 *[38]. The plasticity region genes are speculated to provide the strains with survival benefits in some hosts [25]. This region extends from the ORFs JHP914 to JHP951 in the sequenced strain J99 and is shown to be unstable since some genes within the zone are lost during subsequent infections or laboratory passages [25]. It is now evident from partial sequencing of a Peruvian isolate that this plasticity region might encode yet another type IV secretion system [39] and that the 2 sequenced genomes carry incomplete sets of genes corresponding to this cluster. All the English strains we looked at were similar to either strains 26695 and J99 in that none of them harbored a complete plasticity region cluster as shown recently for a Peruvian strain [39] (data not shown). However, any role of such a putative secretory system is still enigmatic due to lack of correlation of its intactness or abrogation with disease, although, some of the ORFs in the plasticity region have been shown to be associated with a particular disease outcome [25,40]. For English strains, we analyzed seven ORFs from the plasticity region cluster, of which HP986 was strain 26695 specific, while others excluding HP912 were J99 specific. The ORF HP912, the predicted cell division protein/septum formation protein (*fts*A) was PCR amplified in 93.5 % strains. JHP931, a predicted DNA topoisomerase I was amplified in 51%. None of the strains had the same regions of the plasticity zone deleted.

Our phylogenetic analyses based on the FAFLP markers showed a star like distribution on an unrooted neighbor-joining network. Four clusters were evident and the largest cluster was populated mainly by English isolates. Further, affinity to Irish and Indo-European genogroups was also observed. These observations were also substantiated by other slow evolving markers such as ERIC and REP sequences. This denotes stable associations among the genogroups. Similarly, cluster analysis of *bab*B and *glm*M genes also revealed close associations within the European strains and with the Indian strains. However, homologies with East Asian and Amerindian strains were most noteworthy and were comparable to those shown by Irish strains [41]. This reflects ancient genetic events and possible oriental influences on the evolution of *H. pylori *in the English population. Such kinds of non-random genetic links of *H. pylori *may be helpful in understanding evolution of this organism and its clinical consequences in different parts of the world. These findings are in accordance with a recent study that demonstrated that Indian and European *H. pylori *isolates grouped in the same subpopulation and that East Asian and a subset of European isolates share an ancestral relationship and diverged from each other recently [27,42]. The Asian strains, however, were distinctly separated from the European and western strains based on the *cag*A gene sequences except for a few strains that show remote similarity to the East Asian gene pool. We found only a single English strain (N115) that diverged significantly towards the Asian cluster and was recovered from a patient of Indo-Pakistani origin and thereby denotes contribution from the Asian gene pool.

## Conclusion

In summary, our study demonstrated certain distinctive genetic features of the *H. pylori *gene pool in England based on genotypes of virulence genes and neutral markers. Important among these features is the genetic affinity towards East Asian strains. This is also probably the first comprehensive study on detailed, multilocus and multi method genotyping of *H. pylori *from England or elsewhere. The genomic profiles generated in this study may be useful for electronic archiving and retrieval for inter-laboratory comparison and are suitable for storage in epidemiological databases for comparative analyses. However, it will be necessary to analyze additional representative strains, especially from other European populations. Also, our study has largely been an examination of a specific (peptic ulcer disease) group of patient isolates and may not be reflective of other patient isolates from different disease stages in England. Future studies are therefore clearly needed to involve other disease specific strain groups. Further characterization of associations of such informative loci as we examined, in the gene pool of diverse strain groups and with varied disease spectrum may lead to newer insights into the mechanisms of *H. pylori *colonization, and virulence in different hosts.

## Methods

### Bacterial DNA preparations

Sixty-six DNA preparations using Nacl-CTAB method [43] from English *H. pylori *strains were obtained from the strains corresponding to patients reporting at the Queen's Medical Centre, Nottingham, UK. These strains were recovered from patients diagnosed with ulcer disease, having either current ulceration, past ulceration, evidence of scarring at endoscopy or erosions at endoscopy. More than half of these patients were taking acid suppression therapy. These strains were mainly from ethnic English people, although a few were from people originally from Russia (N90), China (N99), South Asia (N105, N115, N131) and Italy (N106) who had settled in the UK. Strains from other countries were taken from our international collection of genomic DNAs provided by our collaborators. Among these are strains from Spain (HupB, n = 7), Ireland (Ire, n = 14), Japan (Hu, n = 10), Peru (Sjm, n = 6), Sardinia (Sard, n = 2), India (MS, n = 1; L, n = 10; BJ, n = 1), Bangladesh (n = 2), Holland (n = 2), and S. Africa (R, n = 8).

### Molecular genotyping and sequencing

Amplification of candidate gene loci including *oip*A, *bab*B, *vac*A middle region, *cag*A and *glm*M genes were carried out as described previously [44,31,41]. Purified PCR amplified products (QIAquick Gel extraction kit) were sequenced using the ABI Prism Big Dye Terminator Cycle Sequencing Reaction Kit (Applied Biosystems, Foster City, USA) in an ABI 3100 automated DNA sequencer.

The *ice*A allele status was determined using oligonucleotide primers mentioned elsewhere [44]. The *cag *A, *cag *E and the *cag *T genes within the *cag *PAI were detected using 4 pairs of primers as mentioned earlier [7]. Analysis of rearrangements of the motifs at the right end of the *cag*A gene and towards the 3' end of the glutamate racemase gene (*glr*) were performed with seven different sets of primers as described previously [31]. PCR primers and procedures used for evaluating the presence of the plasticity region ORFs HP 912, JHP 926, JHP 931, JHP 944, JHP 945, JHP 947 and HP 986 have been described elsewhere [25]. The annealing temperatures for the ORFs HP912, JHP 926 and JHP 944 were standardized to 59, 57 and 66°C respectively for 1 min, followed by an extension at 72°C for 1 minute.

Consensus sequence for each sample was generated using Genedoc (version 2.6.002). Clustal X (version 1.81) was used to align these sequences and dendrograms representing the genetic relationships between strains were generated using Treeview (version 1.6.6). Frame status of the *oip*A gene was analyzed using Lasergene software (DNAStar Inc. USA). Genetic diversity of the *cag*A sequences of all the representative isolates tested from English patients were compared to other records from Genbank [China47 (AJ252985), Hong Kong81 (AF198486), Hong Kong77 (AF198485), Japan 54 (AF198484), S. Africa19 (AF198470), Gambia 4659 (AF198468), Gambia4797 (AF198469), Peru24C (AF198473), Peru34B (AY18476), Guatemala88 (AF198472), India18A (AF202224), India19A (AF202225) DH102 (AY169292), DH200 (AY169294), Dutch107 (AJ252963).]. These Genbank sequences were also used along with sequences from English strains for the phylogenetic tree construction.

### Whole genomic fingerprinting and genotyping

Whole genome fingerprinting based on FAFLP genotyping was done as described previously [43,45]. Briefly, the profiling of whole genome micro-restriction fingerprints with *Eco*RI/*Mse*I enzymes using fluorescence tagged primer pairs *Eco*RI+A/*Mse*I+0 and *Eco*RI+G or A / *Mse*I+0 was performed. The PCR amplified fragments for each of the strains were then subjected to electrophoretic separation on a 5% acrylamide gel and scoring of the fluorescent markers was done using an automated DNA analysis workstation (ABI Prism 3100 DNA sequencer).

The PCR methods for the ERIC fingerprinting technique has been previously described [26]. The REP based typing procedure involved primers for amplifying unique DNA sequences between the two REP signatures [46]. All the gel images corresponding to ERIC and REP PCRs were analyzed using the Quantity 1.0 software in a gel documentation system (Bio-Rad, USA). These images were then uploaded into Diversity 2.2.0 database (Bio-Rad, USA). Band sizes, band attributes and standard molecular weights were assigned alongside the molecular weight markers. Cluster analysis of DNA profiles was conducted on the basis of fingerprint characteristics. Based on the data for the presence or absence of 3–15 different DNA fragments in the fingerprints of strains of *H. pylori*, a binary data matrix was created. Overall similarity between the pairs of strains was calculated from the binary data matrix using the simple matching dice coefficient. The resulting similarity matrix was used for cluster analysis by the unweighted paired group method with arithmetic averages (UPGMA) to generate trees.

### Data archiving and genome wide comparisons

All the data obtained through candidate gene sequencing and DNA profiling was deposited in the genoBASE *pylori *database . The genoBASE *pylori *server was queried for genome wide comparisons. The *cag *PAI rearrangement profiles and *cag *A – *glr *motif types were also compared to existing records in the database.

## Authors' contributions

FK, MAH and IA did all the PCR based genotyping and sequencing. SS and MD carried out phylogenetic analyses. KRR and AAM provided informatics support and comparative analyses on AmpliBASE server. AAK provided isolates from India and provided some infrastructure support for microbiology work. LAS contributed to the editing of the manuscript and partly supervised the study as a collaborator under the *H. pylori *evolutionary genomics interest group. NA conceived, designed and supervised the study, compiled and edited the manuscript and provided overall supervision and leadership.

## Supplementary Material

Additional file 1 Click here for file
