# Supplementary material for: Comparative genomics of Helicobacter pylori isolates recovered from ulcer disease patients in England
Source: BMC Microbiol. 2005 May 25;5:32. doi: 10.1186/1471-2180-5-32 (PMC1180443; doi:10.1186/1471-2180-5-32)
Supplement: Additional file 1 [file 1471-2180-5-32-S1.doc]

| Sample ID | ***Cag* PAI** | | | | ***Cag* RJ motif type** | ***Vac* allele** | ***Ice* allele** | ***Glm*M** | ***Oip*A frame status** | ***Oip*A CT repeat pattern** | ***Bab*B** | Plasticity region ORFs | | | | | | | **Ethnicity** |
| --- | --- | --- | --- | --- | --- | --- | --- | --- | --- | --- | --- | --- | --- | --- | --- | --- | --- | --- | --- |
|  | **AF1** | **AF2** | **E** | **T** |  |  |  |  |  |  |  | **HP986** | **JHP947** | **JHP931** | **JHP926** | **JHP912** | **JHP944** | **JHP945** |  |
| N 1 | 0 | 1 | 1 | 1 | IIIa | s1m2 | iceA1 | 1 | On | 7 | 1 | nd | nd | nd | nd | nd | nd | nd | UK |
| N 3 | 0 | 1 | 0 | 1 | IIIb | s1m1a | iceA1 | 1 | On | 6 | 1 | 1 | 0 | nd | nd | nd | nd | nd | UK |
| N 4 | 1 | 1 | 0 | 0 | * | s1m2 | iceA1 | 1 | Off | 7 | 1 | 1 | 0 | nd | nd | nd | nd | nd | UK |
| N 8 | 0 | 1 | 0 | 1 | Ia | s1m1a | iceA2 | 1 | On | 8 | 1 | 0 | 0 | 0 | 0 | 1 | 0 | 1 | UK |
| N 9 | 0 | 1 | 1 | 1 | IIIa | - | iceA1 | 1 | Off | 5 | 1 | nd | nd | nd | nd | nd | nd | nd | UK |
| N 10 | 0 | 1 | 1 | 1 | Ia | s1m2 | iceA1 | 1 | Off | 7 | 1 | 0 | 0 | 1 | nd | nd | nd | 0 | UK |
| N 11 | 1 | 1 | 1 | 1 | Ib | s1m2 | iceA2 | 1 | nd | nd | 1 | 0 | nd | nd | nd | nd | nd | nd | UK |
| N 16 | 1 | 1 | 1 | 1 | Iib /IV | - | iceA2 | 1 | On | 5+2 | 1 | 1 | 1 | 0 | 1 | 1 | 0 | 1 | UK |
| N 17 | 0 | 1 | 1 | 1 | II | - | iceA2 | 1 | On | 6 | 1 | 1 | 0 | 0 | 0 | 1 | 0 | 0 | UK |
| N 19 | 1 | 1 | 1 | 1 | Ib / III | s1m1a | iceA1 | 1 | Off | 6 | 0 | 0 | 0 | 0 | 1 | 1 | 0 | 0 | UK |
| N 20 | 1 | 1 | 0 | 0 | * | s1m2 | iceA1 | 1 | Off | 7 | 1 | 0 | nd | nd | nd | nd | nd | nd | UK |
| N 21 | 0 | 1 | 0 | 1 | * | s2m2 | iceA2 | 1 | On | 6 | 1 | 0 | 0 | nd | nd | nd | nd | nd | UK |
| N 22 | 0 | 1 | 0 | 1 | II / IIIa | s1m2 | iceA1/ A2 | 1 | Off | 6 | 1 | 1 | 0 | 0 | 0 | 1 | 0 | 1 | UK |
| N 24 | 1 | 1 | 0 | 0 | * | s2m2 | iceA2 | 1 | Off | 3+2 | 1 | 1 | 0 | 0 | 0 | 1 | 0 | 1 | UK |
| N 29 | 0 | 1 | 0 | 1 | IVb | s1m1a | iceA2 | 1 | On | 6 | 0 | 0 | 0 | 0 | 0 | 1 | 0 | 0 | UK |
| N 30 | 0 | 1 | 1 | 1 | Ib | s1m1a | iceA1 | 1 | On | 6 | 1 | 1 | 0 | 0 | 0 | 1 | 0 | 0 | UK |
| N 31 | 0 | 1 | 1 | 1 | IIIa | s1m2 | iceA2 | 1 | On | 6 | 1 | 1 | 0 | 1 | 0 | 1 | 0 | 1 | UK |
| N 32 | 0 | 1 | 0 | 0 | Ib | s1m2 | - | 1 | Off | 9 | 1 | 0 | 0 | 0 | 0 | 1 | 0 | 0 | UK |
| N 35 | 0 | 1 | 1 | 1 | Ia | s1m2 | iceA1 | 1 | On | 6 | 1 | 0 | 0 | 0 | 0 | 1 | 0 | 0 | UK |
| N 38 | 1 | 1 | 1 | 1 | Ia | s1m2 | iceA1 | 1 | Off | 6 | 1 | 0 | 0 | 1 | 0 | 1 | 0 | 0 | UK |
| N 39 | 1 | 1 | 1 | 1 | IVb | s1m2 | iceA2 | 1 | On | 6 | 1 | 0 | 0 | 0 | 0 | 1 | 0 | 0 | UK |
| N 40 | 1 | 1 | 1 | 1 | Ia | s1m2 | iceA1 | 1 | On | 6 | 0 | 1 | 0 | 0 | 0 | 1 | 0 | 0 | UK |
| N 43 | 1 | 1 | 1 | 1 | IIIa | s1m2 | iceA2 | 1 | On | 9 | 1 | 1 | 0 | 1 | 0 | 1 | 0 | 0 | UK |
| N 47 | 1 | 1 | 1 | 1 | Ib | s1m1a | iceA1 | 1 | On | 6 | 1 | 0 | nd | 0 | 1 | 1 | 1 | 1 | UK |
| N 49 | 1 | 1 | 1 | 1 | IIIa | s1m2 | iceA2 | 1 | Off | 5 | 1 | 0 | 1 | 0 | 1 | 1 | 1 | 0 | UK |
| N 51 | 1 | 0 | 1 | 1 | Ia | s1m1a | iceA1 | 1 | On | 1 | 1 | 0 | 0 | 0 | 1 | 1 | 0 | 0 | UK |
| N 52 | 1 | 1 | 1 | 1 | II | s1m1a | iceA1 | 1 | Off | 10 | 1 | 0 | 1 | nd | 1 | 1 | 1 | 1 | UK |
| N 54 | 0 | 1 | 1 | 1 | IIIa | s1m1a | iceA1 | 1 | On | 6 | 1 | 1 | 0 | nd | nd | nd | nd | nd | UK |
| N 56 | 1 | 0 | 0 | 1 | II | s1m1c | iceA2 | 1 | On | 8 | 1 | 1 | 0 | 1 | 0 | 1 | 0 | 1 | UK |
| N 62 | 1 | 1 | 0 | 0 | * | s1m2 | iceA1 | 1 | Off | 7 | 0 | nd | nd | nd | nd | nd | nd | nd | UK |
| N 66 | 0 | 1 | 1 | 1 | IIIa | s1m2 | iceA1 | 1 | On | 9 | 1 | 0 | 0 | nd | nd | nd | nd | nd | UK |
| N 71 | 1 | 1 | 1 | 1 | II | s1m1c | iceA2 | 1 | On | 8 | 0 | 1 | 0 | 1 | 0 | 0 | 0 | 0 | UK |
| N 73 | 1 | 0 | 0 | 1 | * | s2m2 | iceA1 | 1 | Off | 5 | 1 | 0 | 0 | 0 | 1 | 1 | 0 | 1 | UK |
| N 78 | 0 | 1 | 1 | 1 | Ib | s1m1a | iceA2 | 1 | On | 8 | 0 | 0 | 0 | nd | nd | nd | nd | nd | UK |
| N 79 | 1 | 1 | 1 | 1 | IIIa | s1m1a | iceA1 | 1 | On | 6 | 0 | 1 | 1 | 0 | 1 | 1 | 1 | 1 | UK |
| N 82 | 1 | 1 | 0 | 1 | * | s1m2 | iceA1 | 1 | On | 7 | 1 | 0 | 0 | 1 | 0 | 1 | 0 | 1 | UK |
| N 84 | 0 | 1 | 1 | 1 | IVb | s1m2 | iceA1 | 1 | nd | nd | 0 | 0 | nd | nd | nd | nd | nd | nd | UK |
| N 85 | 1 | 1 | 1 | 1 | IIIa | s1m2 | iceA2 | 1 | On | 6 | 0 | nd | nd | nd | nd | nd | nd | nd | UK |
| N 87 | 1 | 1 | 0 | 1 | II | s1m2 | iceA2 | 1 | On | 4 | 1 | 0 | 1 | 0 | 0 | 1 | 0 | 1 | UK |
| N 88 | 0 | 1 | 1 | 1 | Ia | s1m2 | iceA2 | 1 | On | 6 | 1 | 1 | 0 | 0 | 0 | 1 | 1 | 1 | UK |
| N 89 | 0 | 1 | 1 | 1 | II | s1m2 | iceA1 | 1 | On | 2 | 1 | 1 | 0 | 1 | 0 | 1 | 0 | 1 | UK |
| N 90 | 1 | 1 | 1 | 1 | IIIa | s1m2 | iceA1 | 1 | On | 9 | 1 | 1 | 0 | 1 | 0 | 1 | 0 | 1 | Russian |
| N 94 | 1 | 1 | 1 | 0 | * | s1m2 | iceA2 | 1 | Off | 7 | 1 | 1 | 0 | 1 | 0 | 1 | 0 | 1 | UK |
| N 95 | 0 | 1 | 1 | 1 | Ia | s1m1a | iceA2 | 1 | Off | 8 | 0 | nd | 0 | 1 | 0 | 1 | 0 | 0 | UK |
| N 97 | 1 | 0 | 1 | 1 | IIIa | s1m2 | iceA1 | 1 | nd | nd | 0 | 1 | 0 | 1 | 0 | 1 | 0 | 0 | UK |
| N 99 | 0 | 0 | 1 | 1 | IIIa | s1m2 | iceA1 | 1 | On | 9 | 1 | 1 | 0 | 1 | 0 | 0 | 0 | 0 | Chinese |
| N 101 | 1 | 0 | 1 | 1 | Ib | s1m2 | iceA2 | 1 | On | 6 | 1 | 0 | 0 | 1 | 0 | 1 | 0 | 0 | UK |
| N 104 | 0 | 0 | 0 | 0 | * | s1m2 | iceA2 | 1 | On | 6 | 1 | 0 | 0 | 1 | 0 | 1 | 0 | 0 | UK |
| N 105 | 0 | 0 | 1 | 1 | Ia | s1m1a | iceA1/ A2 | 1 | On | 8 | 1 | 0 | 1 | nd | nd | nd | nd | nd | Indo-Pakistani |
| N 106 | 1 | 0 | 1 | 1 | Ib | s1m2 | iceA1 | 1 | On | 1 | 0 | 0 | 1 | 1 | 1 | 1 | 0 | 0 | Italian |
| N 108 | 1 | 0 | 1 | 1 | II | s1m1a | iceA1 | 1 | On | 1 | 1 | 0 | 0 | 1 | 1 | 1 | 0 | 0 | UK |
| N 111 | 1 | 0 | 1 | 1 | IIIa | s1m2 | iceA1 | 1 | On | 6 | 1 | nd | nd | nd | nd | nd | nd | nd | UK |
| N 115 | 0 | 0 | 1 | 1 | II | s1 | iceA1 | 1 | On | 6 | 0 | 1 | 0 | 0 | 0 | 1 | 0 | 1 | Indo-Pakistani |
| N 117 | 1 | 0 | 1 | 1 | Ia | s1m1c | iceA1 | 1 | nd | nd | 1 | 1 | 1 | nd | nd | nd | nd | nd | UK |
| N 123 | 1 | 0 | 1 | 0 | * | s1m2 | iceA1 | 1 | Off | 7 | 0 | 0 | 0 | 0 | 0 | 1 | 0 | 1 | UK |
| N 125 | 0 | 0 | 0 | 1 | Ib | s1m2 | iceA2 | 1 | On | 6 | 1 | 1 | 1 | 1 | 1 | 1 | 0 | 0 | UK |
| N 127 | 1 | 0 | 1 | 1 | IIIa | s1m2 | iceA1 | 1 | nd | nd | 0 | 1 | 0 | 1 | 1 | 1 | 0 | 0 | UK |
| N 129 | 1 | 0 | 1 | 1 | IIIa | s1m2 | iceA1 | 1 | On | 5 | 1 | 1 | 0 | 1 | 1 | 1 | 0 | 0 | UK |
| N 131 | 0 | 0 | 1 | 0 | * | s2m2 | iceA2 | 1 | On | 8 | 1 | 1 | nd | nd | nd | nd | nd | nd | Indo-Pakistani |
| N 135 | 0 | 0 | 0 | 0 | Ia | s1m1a | iceA1 | 1 | On | 7 | 1 | 1 | 1 | 1 | 1 | 1 | 0 | 0 | UK |
| N 139a | 1 | 0 | 0 | 1 | IIIa | s1m2 | iceA1 | 1 | On | 9 | 1 | 1 | 0 | 0 | 0 | 0 | 0 | 0 | UK |
| N 139b | 0 | 0 | 1 | 1 | IV | m2 | iceA1 | 1 | On | 8 | 1 | 1 | 0 | 1 | 0 | 1 | 1 | 0 | UK |
| N 141 | 1 | 0 | 1 | 1 | IIIa | s1m2 | iceA2 | 1 | On | 9 | 1 | 1 | nd | 0 | 1 | 1 | 0 | 1 | UK |
| N 151 | 0 | 0 | 1 | 0 | * | s1m2 | - | 1 | Off | 7 | 1 | nd | nd | nd | nd | nd | nd | nd | UK |
| N 155 | 0 | 0 | 0 | 1 | IVb | s1m2 | iceA1 | 1 | Off | 7 | 1 | 0 | nd | 1 | 0 | 1 | 0 | 0 | UK |
| N 193 | 0 | 0 | 1 | 1 | II | s1m2 | iceA1 | 1 | On | 6 | 1 | 1 | nd | nd | nd | nd | nd | nd | NA |

**Table 1: comprehensive data indicating PCR amplification results of genes in English isolates.**

nd- not determined due to lack of DNA; NA- information not available

0, - - PCR negative for the gene; 1- region present; *- None of the motifs were amplified
